# Diagnostic plasma miRNA-profiles for ovarian cancer in patients with pelvic mass

**DOI:** 10.1371/journal.pone.0225249

**Published:** 2019-11-18

**Authors:** Douglas Nogueira Perez Oliveira, Anting Liu Carlsen, Niels H. H. Heegaard, Kira Philipsen Prahm, Ib Jarle Christensen, Claus K. Høgdall, Estrid V. Høgdall

**Affiliations:** 1 Department of Pathology, Herlev Hospital, University of Copenhagen, Herlev, Denmark; 2 Department of Autoimmunology and Biomarkers, Statens Serum Institut, Copenhagen, Denmark; 3 Department of Congenital Disorders, Statens Serum Institut, Copenhagen, Denmark; 4 Department of Gynaecology, Juliane Marie Centre, Rigshospitalet, University of Copenhagen, Copenhagen, Denmark; Brigham and Women's Hospital, UNITED STATES

## Abstract

**Background:**

Ovarian cancer is the fifth most common cancer in women worldwide. Moreover, there are no reliable minimal invasive tests to secure the diagnosis of malignant pelvic masses. Cell-free, circulating microRNAs have the potential as diagnostic biomarkers in cancer. Here, we performed and validated a miRNA panel with the potential to distinguish OC from benign pelvic masses.

**Methods:**

The profile of plasma microRNA was determined with a panel of 46 candidates in a discovery group and a validation group, each consisting of 190 pre-surgery plasma samples from age-matched patients with malignant (n = 95) and benign pelvic mass (n = 95), by real time RT-qPCR.

**Results:**

Four up-regulated (miR-200c-3p, miR-221-3p, miR-21-5p, and miR-484) and two down-regulated (miR-195-5p and miR-451a) microRNAs were discovered. From those, miR-200c-3p and miR-221-3p were further confirmed in a validation cohort. A combination of these 2 microRNAs together with CA-125 yielded an overall diagnostic accuracy of AUC = 0.96.

**Conclusions:**

We showed consistent plasma microRNA profiles that provide independent diagnostic information of late stage OC.

## Introduction

Ovarian cancer (OC) is the major cause of cancer death among women globally [1]. With approximately 324 new OC cases and 124 borderline tumours every year, Denmark has one of the highest incidence rates of OC in the world, with an estimate of 14 cases per 100,000 in the recent years [2, 3]. Due to its asymptomatic features, OC is typically diagnosed at late stage (FIGO III-IV), in 65% of all cases, impacting on a poor 5-year overall survival of less than 50% [2]. Surgical debulking and precise surgical staging are essential for good OC management, where an optimal preoperative differentiation between OC and benign pelvic masses is critical to improve survival [4, 5]. However, those procedures are reserved to specialised tertiary gynaecologic oncology centres. Moreover, due to the high unspecificity of the current diagnostic markers, only about 20–25% of patients with benign pelvic mass will be diagnosed with OC [5, 6], underscoring the lack of reliable tests to secure the diagnosis. Therefore, the relative high prevalence, late diagnosis and consequent poor prognostics in OC are still a critical clinical challenge with unmet needs.

For the past two decades micro-RNAs (miRNAs) have emerged as promising diagnostic and prognostic biomarkers. MiRNAs are small non-coding RNAs, ranging between 19-25nt which can regulate gene expression by binding to their complementary target messenger RNA (mRNA) [7]. Depending on the binding region of the miRNA to their target mRNA, it can lead mainly to suppression or degradation of its target [8, 9]. Despite their ubiquitous nature miRNAs are tissue-specific, and their expression has also been associated with different stages of OC and clinical outcome [7, 10, 11]. Additionally, circulating miRNAs are highly stable in the body fluid, such as plasma, serum, urine, saliva, cerebrospinal fluid, amniotic and follicular fluids, presenting as a versatile non-invasive indicator for a wide range of cancers [12–14]. Thus, they may serve as potential biomarkers offering rapid and easy tests, and eliminating the necessity of performing invasive procedures, such as biopsies [15–18].

Early studies on genome-wide comparison of miRNA expression between OC and normal ovary tissue have found a set of miRNAs to be differentially represented [10]. More recently, a limited number of studies have suggested the existence of differential expression of circulating miRNA between healthy women and patients with pelvic masses. However, such studies were performed on very small sample sizes and did not focus on differentiating benign from malignant pelvic mass [7, 13, 19].

In this study, using an initial discovery cohort of 190 plasma samples from patients with pelvic masses, classified as benign control (n = 95) and malignant (n = 95), we investigated a panel of 46 miRNAs previously reported to be associated with ovarian and other cancers. We further validated our findings on an independent cohort with benign pelvic masses and late stage OC (FIGO III-IV).

## Materials and methods

### Patients and samples handling

All patients in the current study were recruited from the Pelvic Mass study, initiated in September 2004 and finalized in August 2007. Patients were referred to surgery for suspected pelvic tumor, following PET/CT exams within two weeks prior to procedure. Furthermore, all patients were subjected to abdominal and vaginal ultrasound, and serum CA-125 level and menopausal state assessments. Exclusion criteria were pregnancy, previous cancer or borderline tumour, patient unable to understand any information about the study, and cancellation of planned surgery because of no suspicion of pelvic disease after further examinations. All tissue specimens obtained during the exploratory surgery were examined by a specialized pathologist. All patients were registered in the Danish Gynaecologic Cancer Database (DGCD), which is a national compulsory research and quality on-line database. All proceedings were carried out according to the precepts of the Declaration of Helsinki, including written informed consent from all participating patients. The study has been approved by the Danish National Committee for Research Ethics, Capital Region (approval codes KF01-227/03 and KF01-143/04).

Blood samples were secured less than 14 days before surgery. They were collected as described previously [20], and all handling procedures were accomplished within 6 h.

### RNA purification and RT-qPCR

RNA purification and reverse transcription followed by quantitative PCR (RT-qPCR) were performed as previously described [19]. Briefly, total RNA was purified from 380 plasma samples (100 μl/sample) using Norgen Total RNA Purification Kit (Norgen Biotek Corp. Ontario, Canada), with addition of 10mM DTT. As a technical control for further RT-qPCR analysis, *C*. *elegans* synthetic cel-mir-54 and cel-mir-238 (TAG Copenhagen A/S) were mixed to the lysis buffer prior to adding to each sample. Purified RNA was stored at -20°C until use. Reverse transcription was performed on a panel of 46 miRNAs (TaqMan microRNA Reverse Transcription Kit), followed by preamplification (TaqMan PreAmp master mix), and RT-qPCR using TaqMan miRNA assays (Applied Biosystems, Foster city, CA, USA). For the qPCR, we used a Fluidigm BioMark system (Fluidigm Corp. USA) allowing for a duplicate assay in one operational run. Four 96.96 gene expression chips (Fluidigm Corp. USA) were used. The panel of 46 miRNAs is described on S1 Table.

### Data handling

For data analysis the auto detectors settings were chosen for signal detection threshold. Average raw Cq values above 35 were excluded from the data set. Each remaining average Cq value was normalized by subtracting the average of Cq of cel-miR-54 and cel-miR-238 for that particular sample, yielding the -ΔCq (average Cq of cel-miR-54 and cel-miR-238 –average Cq of hsa-miR). The -ΔCq values were further normalized with the average -ΔCq of all detected miRNAs in all samples to further correct for variations in total input RNA. In the end, we used the average -ΔCq of 23 miRNAs to subtract in the discovery cohort and 29 miRNAs in the validation cohort. These double-normalized expression values were used for all statistical analyses.

### Statistical analysis

Univariate analysis was performed on all miRNAs in the discovery cohort, identifying any with a p-value < 0.05. The statistical analysis approach was logistic regression modelling for the probability of malignant pelvic mass versus benign. The miRNAs were then subjected to a multivariate analysis on this cohort in order to select for further analysis. To account for missing values, multiple imputation [21] was then employed, assuming missing at random (MAR). The multivariate analysis was performed with 25 imputations for those miRNAs that passed the univariate analysis, selecting for candidates with p<0.005. Model fitting was assessed using martingale residuals and the Hosmer-Lemeshow test [22]. The final analysis included the selected miRNAs and clinical covariates assessed in the validation cohort. Results are presented by odd ratios (OR) with 95% confidence intervals (CI) and the area under the receiver operating characteristic curve (AUC). In addition, the positive predictive values (PPV), as well as the negative predictive values (NPV), were calculated for cutoffs corresponding to 80% sensitivity.

Statistical calculations were done using SAS (SAS Institute, Cary, N.C., USA, v9.4) and R (R Development Core Team, Vienna, Austria) [23]. An overview of data handling and statistical analysis is shown on S1 Fig.

## Results

### Clinicopathological characteristics of patients

In a total of 380 patients, 190 were diagnosed with benign pelvic masses, and 190 with malignant condition, categorised according to their respective FIGO stage and histological classification. Samples with not enough plasma material or poor RNA quality were further excluded in order to avoid technical outliers (14 OC and 13 benign cases). CA-125 levels and body mass index (BMI) were also measured. Patient demographics are shown on Table 1.

**Table 1:** Demographics of pelvic mass patients on the two cohorts.

	Discovery cohort (n = 190)	Validation cohort (n = 190)
**Benign pelvic mass**	95	95
Age, median, (range), y	66 (28–90)	51 (42–86)
Histology		
Serous Cystadenoma	31	19
Mucinous Cystadenoma	32	6
Endometriosis	2	27
Teratoma	0	1
Fibroma/ Leiomyoma*	7	13
Functional/simple/ haemorrhagic/paratubal ov. cysts**	15	29
Other	8	0
CA-125 median (range), U/ml	23 (2–1906)	
**Ovarian cancer**	95	95
Age, median (range), y	64 (41–88)	66 (17–89)
FIGO Stage		
I	5	0
II	8	0
III	61	72
IV	21	23
Histology		
Serous adenocarcinoma	80	82
Mucinous adenocarcinoma	5	1
Endometrioid adenocarcinoma	4	2
Clear cell neoplasms	4	3
Carcinosarcoma	1	1
Non-differentiated carcinoma	1	2
Sex-chord tumour	0	1
Unknown	0	1
CA-125 median (range), U/ml	544 (29–12310)	

*Including serous adenofibroma

**Including dermoid cysts.

All OC cases in this study were traced by the Danish Central Population Register (CPR) and date of death or emigration were registered until January 11th, 2011. In addition, information about treatment (surgery and chemotherapy) and cause of death was retrieved from the DGCD database. In total, 127 patients completed the follow-up period for this study–a total of 73 OC patients died from OC (median follow-up time: 18 months, range: 1–68) and 54 patients were still alive (median follow-up time: 56 months, range: 38–75).

### Discovery of differential miRNA modulation in malignant cases

The profile of plasma miRNA was investigated with a panel of 46 miRNAs (S2 Table). The panel was assembled based on miRNA known to be associated with OC or other types of cancer. By univariate analysis we found 4 miRNAs to be significantly up-regulated–hsa-miR-21-5p (OR = 2.15; p = 0.0046), has-miR-200c-3p (OR = 1.88; p = 0.0001), hsa-miR-221-3p (OR = 2.43; p = 0.0006), and hsa-miR-484 (OR = 1.38; p = 0.0181)–and 2 miRNAs down-regulated–hsa-miR-195-5p (OR = 0.73; p = 0.0071) and hsa-miR-451a (OR = 0.68; p = 0.0046)–in patients with malignant pelvic mass compared to patients with a benign ovarian tumour (Table 2).

**Table 2:** Candidate miRNAs found on the ovarian cancer discovery cohort.

miRNA	OR	p-value	AUC
CA-125	2.40	<0.0001	0.92
hsa-miR-200c-3p	1.88	<0.0001	0.78
hsa-miR-221-3p	2.43	0.0006	0.65
hsa-miR-195-5p	0.73	0.0071	0.63
hsa-miR-21-5p	2.15	0.0046	0.63
hsa-miR-451a	0.68	0.0046	0.62
hsa-miR-484	1.38	0.0181	0.63

OR: odds ratio. AUC: area under the curve. All p-values < 0.05 were considered as statistically significant.

**Fig 1:**
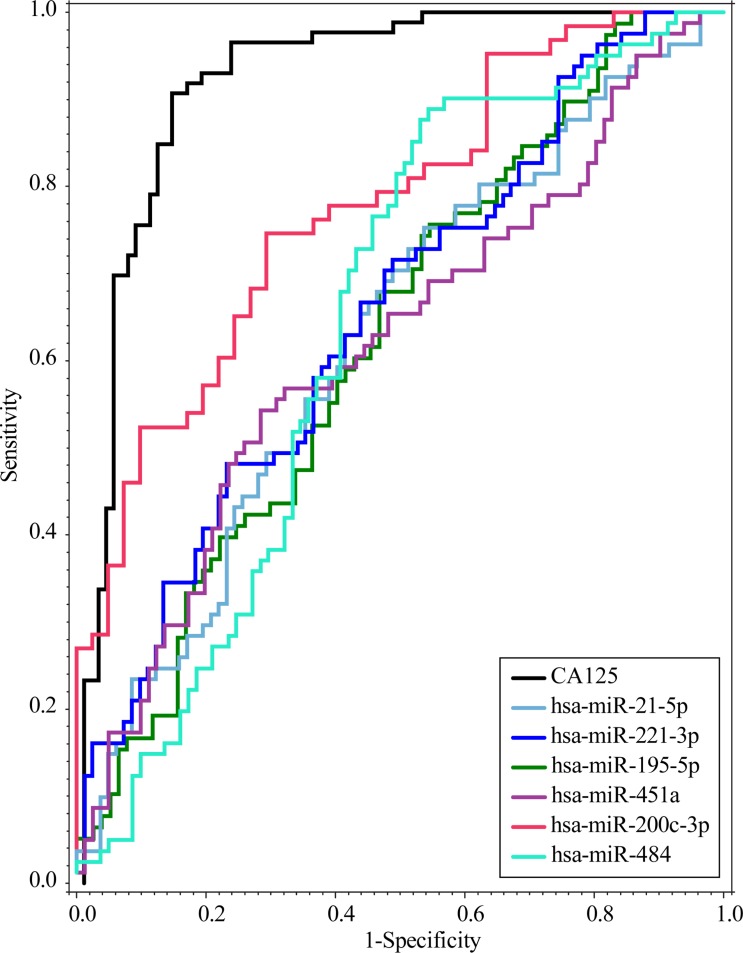
Predictive efficiency of the 6 miRNAs found on the discovery cohort. Receive Operating Characteristic (ROC) curves were performed for each miRNA, and CA-125.

We further investigated the ability of all candidate miRNAs to discriminate between benign and malignant cases by individually calculating the Receiver Operating Characteristic (ROC) curves, including for CA-125. Among the 6 candidates, hsa-miR-200c-3p showed the highest predictive efficiency (AUC = 0.78), with an OR = 1.88 (95% CI:1.42–2.49), followed by hsa-miR-221-3p (AUC = 0.65), hsa-miR-195-5p (AUC = 0.63), hsa-miR-21-5p (AUC = 0.63), hsa-miR-484 (AUC = 0.63), and hsa-miR-451a (AUC = 0.62) (Fig 1). CA-125 measurements had the highest index, with AUC = 0.92.

### A combination of miRNAs and CA-125 levels might increase diagnostic accuracy of OC

To confirm our findings, we implemented further statistical analysis on our discovery cohort. To ensure that such findings were not biased in the first place by an incomplete set of data in our discovery cohort, multiple imputation with 25 iterations was done. Multivariate analysis on the imputed dataset was performed including all 6 candidate miRNAs in order to explore association between them and the presence of malignant pelvic mass. We found 2 miRNAs–hsa-miR-200c-3p and hsa-miR-221-3p –to be strongly associated with OC (p< 0.0001 for the logistic model). The discrimination for the combination of these 2 miRNAs, showed AUC = 0.75 ± 0.04.

**Fig 2:**
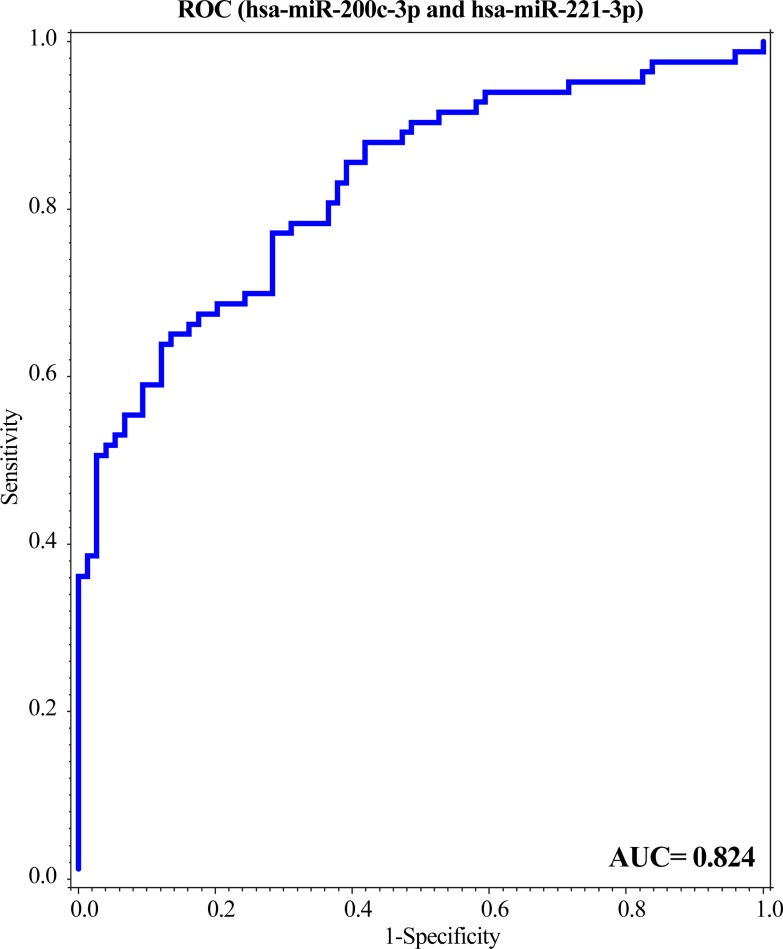
The validated miRNAs were capable to identify ovarian cancer with a high predictive efficiency. A ROC curve was performed for the combination of miR-200c-3p and miR-221-3p validated by an independent cohort.

The multivariate model from the discovery cohort was then applied to the validation cohort demonstrating an increased accuracy efficiency (AUC = 0.82), with a sensitivity of 80% and 64% specificity (Fig 2) which corresponded to a PPV of 71% and NPV 73%.

**Fig 3:**
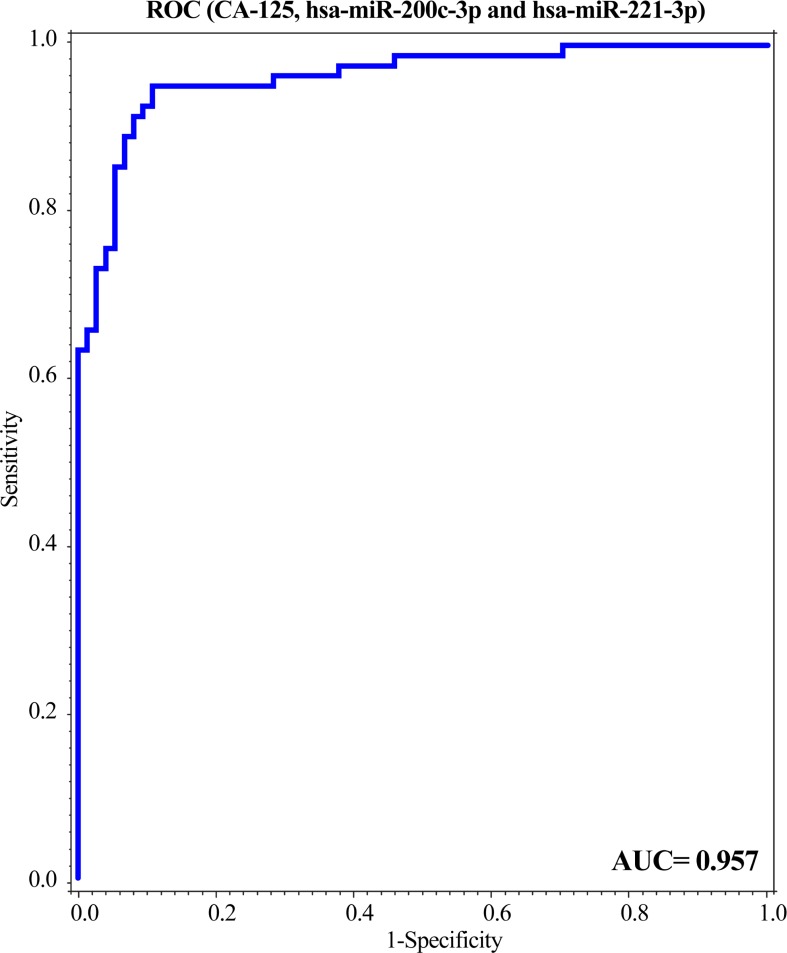
The predictive efficiency of miR-200c-3p and miR-221-3p combined with CA-125 improves ovarian cancer detection. A ROC curve was performed for the combination of both miRNAs together with CA-125.

Considering that CA-125 is currently used in the clinic for OC management, it alone presents as a very poor predictive marker, with limited specificity. Here, we tested the combination of CA-125 with hsa-miR-200c-3p and hsa-miR-221-3p together for its predictive ability. The performance of CA-125 in the validation cohort showed an AUC = 0.94, whilst the combination of both miRNAs and CA-125, as well as age, had a modest improvement at a high level, AUC = 0.96. However, the contribution of the miRNA’s was statistically significant (p = 0.004) with excellent discrimination, presenting the substantial overall predictive performance, with sensitivity = 80% and specificity = 95%, and PPV = 94% and NPV = 80% (Fig 3; S3 Table).

Noteworthy, the number of serous adenocarcinoma subtype samples is remarkably higher in both of cohorts compared to the other subtypes. However, the expression levels of both hsa-miR-200c-3p and hsa-miR-221-3p is similar among all subtypes, except for the endometrioid adenocarcinoma subtype (p = 0.038), likely due to the small sample size (n = 4) and the large standard deviation in that subtype (S2 Fig). In addition, further statistical analysis considering only serous adenocarcinoma did not show any increase on the performance of those markers (AUC = 0.95).

## Discussion

New biomarkers for OC are needed in order to improve diagnosis in patients presenting with pelvic masses. In recent years it has been discovered that miRNAs can be abundantly found in circulating blood, present as a highly stable form due to its binding to protein complexes or packed into extracellular vesicles, making them resistant to degradation [24, 25]. Thus, miRNAs have the potential as a non-invasive diagnostic and prognostic marker [15–18]. Previously, only a limited number of studies have been performed in a small sample of patients with reported pelvic masses (benign and malignant), but without further validation [7, 13]. None has evaluated and validated the association between microRNA expression patterns in plasma in large groups of women admitted for symptoms of a pelvic mass.

Using presurgical plasma samples from 380 women enrolled in the Danish Pelvic Mass study, we divided the quantitative profiling of 46 miRNAs into two groups: (I) a discovery group of 190 women either harbouring early to late stage OC (n = 95) or benign ovarian conditions (n = 95); and (II) an independent validation group of an additional 190 women representing benign ovarian tumours and late stage OC. We demonstrated that the discovered candidate miRNAs markers could be validated on an independent cohort. Moreover, we showed that plasma-derived miRNA profiles can provide independent diagnostic information in at least late stage cases.

In this study the differential profile of plasma miRNA was investigated by RT-qPCR technology in patients with malignant and benign pelvic masses. The panel of miRNAs was selected based on previous reported association with OC or other cancer types. We found six miRNAs on our discovery cohort (hsa-miR-21-5p, -484, -195-5p, -451a, -200c-3p, and -221-3p,) to be differentially expressed between the two patient groups. Of those, hsa-miR-21-5p is one of the most frequently studied miRNAs in cancer and might serve as a potential biomarker for human cancer diagnosis [26]. Consistent with our finding, the upregulation of hsa-miR-21 in OC has been reported in several studies [14, 27, 28]. The relevance of hsa-miR-484 is still controversial, and it has been found in different types of cancer or even in different materials of same type of cancer [29]. In our study we found it consistently up-regulated in patients with malignant pelvic masses. Among the down-regulated miRNAs, we found hsa-miR-195-5p expression to be decreased in our validation cohort. Interestingly, this miRNA is also found in low amounts in breast cancer and has been described as a potential biomarker for chemoresistance of triple-negative breast cancer cases [30, 31]. However, this is the first time that it is reported in OC. We also found the expression of hsa-miR-451a to be decreased in malignant pelvic mass cases in comparison to the benign group. This miRNA has been previously included in a panel of serum markers for OC, but similar to our results its association was not further evidenced on their validation panel [32].

To further validate our findings, we tested our model for miRNAs on an independent cohort comprising benign pelvic mass and late stage OC. We first performed multiple imputation, followed by multivariate analysis and goodness-of-fit model on the validation cohort, in order to test for the accuracy of the model. With the coefficients found on these settings, we applied them to our validation cohort. In agreement with the discovery cohort, hsa-miR-200c-3p and hsa-miR-221-3p were found dysregulated on our validation study. The miR-200 family members are ubiquitous to many types of cancer and believed to be metastasis suppressors, with repressive effects on cell transformation, cancer cell proliferation, migration, invasion and metastasis [33–36]. We investigated the miR-200 family members (miR-200a, miR-200b, miR-200c, miR-141 and miR-429) on our study as well. Consistently, we found only miR-200c-3p significantly up-regulated in patients with malignant pelvic mass. The miR-200 family members have a known putative role in tumor development, and it has been reported that their dysregulation is associated with cell migration and invasion, whilst promoting drug resistance [35, 37]. Such function might be associated with class III β-tubulin (TUBB3), where restoration of miR-200c led to an increase in sensitivity to microtubule-targeting drugs [37]. Additionally, mesenchymal and transcriptional factor genes (such as *NTRK2* and *FN1*, and *ZEB1* and *ZEB2*, respectively) were also confirmed to be miR-200c targets, affecting cell migration and invasion [37, 38]. In a similar manner, hsa-miR-221-3p also presented overexpression in our cohort of OC. This miRNA has also been observed in various advanced cancer type, especially in epithelial cancer [39]. Hong and colleagues have investigated the levels of miR-221 in serum of patients with epithelial OC. MiR-221 was capable to differentiate patients on different FIGO stages and also indicated to be associated with patient overall survival, with higher expression on cases with worst prognosis [40]. Similar to miR-200 family member, miR-221 has been shown to be involved in diverse tumor development pathways. Zhang and colleagues showed that elevated levels of miR-221 are capable to bypass apoptosis and stimulate cell proliferation by targeting a crucial p53-associated apoptosis pathway [41]. MiR-221 can also act as an oncomiR–overexpression of miR-221 was shown to be associated with cell proliferation, colony formation and invasion in prostate cancer cell lines, by targeting the expression of the tumor suppressor ARHI [42].Despite their pervasive nature in various cancer types, we showed here that the combination of hsa-miR-200c-3p and -221-3p as diagnostic markers increases the specificity and sensitivity to detect OC. Furthermore, using a limited but still widely employed indicator for OC, CA-125 levels, together with miR-200c-3p and -221-3p improves the overall diagnostic accuracy even further, increasing its sensitivity and specificity. Nonetheless, considering the small number of early stage cases on our discovery cohort, the performance of such markers was not feasible for those cases alone. Hence, the validation was performed in late stage (FIGO III-IV) cases only.

The association and kinetics of these miRNAs in plasma may be difficult to explain since they may derive from different sources. A hypothesis is that these circulating miRNAs may be released either from cellular degradation of the tumoral tissue, or from extra vesicular particles released by normal healthy or damaged cell types. However, due to its high stability circulating in the body and their tissue specificity, plasma miRNAs may nonetheless serve as valuable biomarkers [15]. Currently there are no universally implemented guidelines for the collection, preparation and extraction of samples, and analytical methods for circulating miRNA analysis. The lack of standardization and implementation of normalization methods is especially challenging [43, 44]. Therefore, caution is warranted in interpreting data from various studies. In this study, we used a relevant population of cases and controls in relatively large sample sets, subjected to stringent statistical analysis. Noteworthy, the samples on this study were handled following the best practice guidelines (BPGs) from Bio- and Genomebank Denmark (www.RBGB.dk).

Taken together, our results indicated that the association of miR-200c-3p, -221-3p and CA-125 was able to improve differentiation between late stage OC and benign cases. Notably, despite resulting on a marginal improvement when compared to CA-125 alone, our model was highly reproducible. Moreover, early diagnosis is of great importance as it is likely to further improve patient overall survival, considering that 5-year survival rate for early stage OC (> 90%) are sensibly higher than for advanced stages (< 30%) [1, 45]. Therefore, the validation with a cohort from early stage OC with our identified plasma-derived miRNA profiles may provide independent diagnostic information in the early stage cases.

## Conclusion

We found that plasma miRNA profiles distinguished patients with late stage OC from benign ovarian conditions consistently in both the discovery and validation cohorts. Moreover, the combination of miRNAs we found in this study and routinely used serum CA-125 levels [46] may improve the diagnosis of the disease in regard to sensitivity. In the future, we seek to explore our findings by further investigating whether those markers are also capable of identifying early stage OC patients. In this way, this approach might show very valuable for OC patient treatment, overall survival and quality of life.

## Supporting information

S1 Table Sequence of the 48 TaqMan miRNA assays. (DOCX)

S2 Table Summary expression of miRNAs on the discovery and validation cohorts (log2). (DOCX)

S3 Table Sensitivity and specificity rates of the markers on the discovery cohort. (DOCX)

S1 Fig Analysis pipeline for both discovery and validation cohorts. (PDF)

S2 Fig MiRNA expression in different histologic types. (PDF)
