# Supplementary material for: Diagnostic plasma miRNA-profiles for ovarian cancer in patients with pelvic mass
Source: PLoS One. 2019 Nov 18;14(11):e0225249. doi: 10.1371/journal.pone.0225249 (PMC6860451; doi:10.1371/journal.pone.0225249)
Supplement: S1 Table — (DOCX) [file pone.0225249.s001.docx]

**S1 Table. Sequence of the 48 TaqMan miRNA assays**

| **TaqMan Assay Name** | **TaqMan Assay ID** | **miRBase ID** | **Mature miRNA sequence** |
| --- | --- | --- | --- |
| hsa-let-7f | 000382 | hsa-let-7f-5p | UGAGGUAGUAGAUUGUAUAGUU |
| hsa-miR-20a | 000580 | hsa-miR-20a-5p | UAAAGUGCUUAUAGUGCAGGUAG |
| hsa-miR-21 | 000397 | hsa-miR-21-5p | UAGCUUAUCAGACUGAUGUUGA |
| hsa-miR-23a | 000399 | hsa-miR-23a-3p | AUCACAUUGCCAGGGAUUUCC |
| hsa-miR-23b | 000400 | hsa-miR-23b-3p | AUCACAUUGCCAGGGAUUACC |
| hsa-miR-25 | 000403 | hsa-miR-25-3p | CAUUGCACUUGUCUCGGUCUGA |
| hsa-miR-26a | 000405 | hsa-miR-26a-5p | UUCAAGUAAUCCAGGAUAGGCU |
| hsa-miR-27a | 000408 | hsa-miR-27a-3p | UUCACAGUGGCUAAGUUCCGC |
| hsa-miR-27b | 000409 | hsa-miR-27b-3p | UUCACAGUGGCUAAGUUCUGC |
| hsa-miR-29a | 002112 | hsa-miR-29a-3p | UAGCACCAUCUGAAAUCGGUUA |
| hsa-miR-92a | 000430 | hsa-mir-92a-3p | UAUUGCACUUGUCCCGGCCUG |
| mmu-miR-93 | 001090 | hsa-miR-93-5p | CAAAGUGCUGUUCGUGCAGGUAG |
| mmu-miR-96 | 000186 | hsa-miR-96-5p | UUUGGCACUAGCACAUUUUUGCU |
| hsa-miR-103 | 000439 | hsa-miR-103a-3p | AGCAGCAUUGUACAGGGCUAUG |
| hsa-miR-106a | 002169 | hsa-miR-106a-5p | AAAAGUGCUUACAGUGCAGGUAG |
| hsa-miR-122 | 002245 | hsa-miR-122-5p | UGGAGUGUGACAAUGGUGUUUG |
| hsa-miR-125b | 000449 | hsa-miR-125b-5p | UCCCUGAGACCCUAACUUGUGA |
| hsa-miR-126 | 002228 | hsa-miR-126-3p | UCGUACCGUGAGUAAUAAUGCG |
| hsa-miR-140-3p | 002234 | hsa-miR-140-3p | UACCACAGGGUAGAACCACGG |
| hsa-miR-141 | 000463 | hsa-miR-141-3p | UAACACUGUCUGGUAAAGAUGG |
| hsa-miR-142-3p | 000464 | hsa-miR-142-3p | UGUAGUGUUUCCUACUUUAUGGA |
| hsa-miR-145-5p | 002278 | hsa-miR-145-5p | GUCCAGUUUUCCCAGGAAUCCCU |
| hsa-miR-148b | 000471 | hsa-miR-148b-3p | UCAGUGCAUCACAGAACUUUGU |
| hsa-miR-152 | 000475 | has-miR-152-3p | UCAGUGCAUGACAGAACUUGG |
| hsa-miR-181b | 001098 | hsa-miR-181b-5p | AACAUUCAUUGCUGUCGGUGGG |
| hsa-miR-182 | 002334 | hsa-miR-182-5p | UUUGGCAAUGGUAGAACUCACACU |
| hsa-miR-191 | 002299 | hsa-miR-191-5p | CAACGGAAUCCCAAAAGCAGCUG |
| hsa-miR-195 | 000494 | hsa-miR-195-5p | UAGCAGCACAGAAAUAUUGGC |
| hsa-miR-199a-3p | 002304 | hsa-miR-199a/b-3p | ACAGUAGUCUGCACAUUGGUUA |
| hsa-miR-200a | 000502 | hsa-miR-200a-3p | UAACACUGUCUGGUAACGAUGU |
| hsa-miR-200b | 002251 | hsa-miR-200b-3p | UAAUACUGCCUGGUAAUGAUGA |
| hsa-miR-200c | 002300 | hsa-miR-200c-3p | UAAUACUGCCGGGUAAUGAUGGA |
| hsa-miR-205 | 000509 | hsa-miR-205-5p | UCCUUCAUUCCACCGGAGUCUG |
| hsa-miR-214 | 002306 | hsa-miR-214-3p | ACAGCAGGCACAGACAGGCAGU |
| hsa-miR-221 | 000524 | hsa-miR-221-3p | AGCUACAUUGUCUGCUGGGUUUC |
| hsa-miR-223 | 002295 | hsa-miR-223-3p | UGUCAGUUUGUCAAAUACCCCA |
| hsa-miR-335 | 000546 | hsa-miR-335-5p | UCAAGAGCAAUAACGAAAAAUGU |
| hsa-miR-346 | 000553 | hsa-miR-346 | UGUCUGCCCGCAUGCCUGCCUCU |
| hsa-miR-372 | 000560 | hsa-miR-372-3p | AAAGUGCUGCGACAUUUGAGCGU |
| hsa-miR-373 | 000561 | hsa-miR-373-3p | GAAGUGCUUCGAUUUUGGGGUGU |
| hsa-miR-378 | 002243 | hsa-miR-378a-3p | ACUGGACUUGGAGUCAGAAGG |
| hsa-miR-424 | 000604 | hsa-miR-424-5p | CAGCAGCAAUUCAUGUUUUGAA |
| hsa-miR-429 | 001024 | hsa-miR-429 | UAAUACUGUCUGGUAAAACCGU |
| mmu-miR-451 | 001141 | hsa-miR-451a | AAACCGUUACCAUUACUGAGUU |
| hsa-miR-484 | 001821 | hsa-miR-484 | UCAGGCUCAGUCCCCUCCCGAU |
| hsa-miR-503 | 001048 | hsa-miR-503-5p | UAGCAGCGGGAACAGUUCUGCAG |
| cel-miR-54 | 001361 | cel-miR-54-3p | UACCCGUAAUCUUCAUAAUCCGAG |
| cel-miR-238 | 000248 | cel-miR-238-3p | UUUGUACUCCGAUGCCAUUCAGA |
