# Supplementary material for: Diagnostic plasma miRNA-profiles for ovarian cancer in patients with pelvic mass
Source: PLoS One. 2019 Nov 18;14(11):e0225249. doi: 10.1371/journal.pone.0225249 (PMC6860451; doi:10.1371/journal.pone.0225249)
Supplement: S2 Table — (DOCX) [file pone.0225249.s002.docx]

**S2 Table. Summary expression of miRNAs on the discovery and validation cohorts (log2)**

|  | **Discovery cohort** | | | | | | **Validation cohort** | | | | | |
| --- | --- | --- | --- | --- | --- | --- | --- | --- | --- | --- | --- | --- |
|  | **Benign (n=82)** | | | **OC (n=86)** | | | **Benign (n=95)** | | | **OC (n=90)** | | |
| **miRNA** | **Median** | **Min.** | **Max.** | **Median** | **Min.** | **Max.** | **Median** | **Min.** | **Max.** | **Median** | **Min.** | **Max.** |
| **hsa-miR-106a-5p** | 0.35 | -2.29 | 4.00 | 0.49 | -2.67 | 2.74 | 0.54 | -2.92 | 2.36 | -0.25 | -3.25 | 2.64 |
| **hsa-miR-199a-5p** | -2.18 | -7.16 | -0.71 | -2.14 | -6.74 | -0.92 | -2.68 | -2.68 | -2.68 | . | . | . |
| **hsa-miR-26a-5p** | -1.32 | -5.48 | 2.54 | -1.33 | -3.48 | 0.59 | -3.16 | -4.90 | -1.77 | -2.98 | -4.68 | -1.07 |
| **hsa-miR-335-5p** | -2.33 | -6.40 | -0.08 | -2.21 | -5.08 | 0.70 | -2.72 | -4.51 | -0.79 | -2.38 | -4.68 | -0.06 |
| **hsa-miR-451a** | 0.67 | -2.09 | 4.62 | -0.02 | -2.16 | 2.79 | -0.50 | -3.40 | 1.70 | -1.30 | -4.03 | 2.02 |
| **hsa-miR-484** | -1.23 | -3.48 | 3.14 | -0.66 | -2.80 | 4.77 | 2.90 | 0.13 | 4.84 | 3.51 | 0.57 | 5.38 |
| **hsa-miR-142-3p** | -0.63 | -2.08 | 1.23 | -0.70 | -2.20 | 1.33 | -0.80 | -2.29 | 0.95 | -0.51 | -3.10 | 1.07 |
| **hsa-miR-191-5p** | 0.54 | -2.64 | 2.64 | 0.51 | -1.53 | 2.75 | 0.00 | -1.62 | 1.65 | -0.21 | -1.85 | 1.51 |
| **hsa-miR-21-5p** | 1.59 | 0.20 | 3.27 | 1.76 | 0.48 | 3.64 | 1.39 | 0.63 | 2.65 | 2.00 | 0.14 | 3.23 |
| **hsa-miR-221-3p** | -0.73 | -3.52 | 0.88 | -0.40 | -1.59 | 1.08 | -0.11 | -2.58 | 1.42 | -0.07 | -4.44 | 0.96 |
| **hsa-miR-223-3p** | 4.12 | -0.47 | 6.36 | 3.93 | 0.59 | 6.11 | 5.52 | 3.17 | 7.31 | 4.98 | 2.96 | 7.34 |
| **hsa-mir-92a-3p** | 1.06 | -0.10 | 2.14 | 0.91 | -0.79 | 2.54 | 1.84 | 0.06 | 2.92 | 1.98 | 0.64 | 3.26 |
| **hsa-miR-93-5p** | -0.31 | -2.57 | 2.94 | -0.32 | -4.07 | 2.22 | -0.84 | -3.32 | 0.61 | -1.74 | -5.73 | 1.02 |
| **hsa-miR-145-5p** | -5.74 | -12.56 | -1.10 | -5.13 | -13.71 | -1.82 | -6.04 | -10.38 | -2.13 | -6.59 | -13.91 | -3.34 |
| **hsa-miR-148b-3p** | -3.82 | -8.28 | -2.13 | -3.96 | -7.37 | -2.44 | -4.38 | -6.23 | -2.27 | -4.15 | -6.77 | -2.55 |
| **hsa-miR-96-5p** | 10.63 | -13.57 | 14.45 | 10.08 | -12.05 | 14.21 | 8.13 | -6.47 | 12.09 | 8.31 | 5.28 | 12.27 |
| **hsa-miR-126-3p** | 0.41 | -2.10 | 2.32 | 0.36 | -2.70 | 1.88 | 0.12 | -1.71 | 2.08 | 0.31 | -2.25 | 1.19 |
| **hsa-miR-141-3p** | -1.53 | -5.66 | 1.64 | -1.73 | -6.24 | 3.20 | -3.27 | -14.23 | 0.41 | -2.77 | -8.15 | 1.27 |
| **hsa-miR-27a-3p** | -0.89 | -2.52 | 0.27 | -0.82 | -3.75 | 0.10 | -1.36 | -2.49 | 0.82 | -1.27 | -3.04 | -0.05 |
| **hsa-miR-346** | -1.13 | -11.33 | 4.44 | -1.40 | -7.80 | 7.30 | -8.96 | -18.20 | -3.73 | -10.09 | -14.53 | -4.29 |
| **hsa-miR-181b-5p** | -3.06 | -5.40 | 0.20 | -3.26 | -5.48 | 2.98 | 4.71 | 0.48 | 7.98 | 5.15 | 1.89 | 9.07 |
| **hsa-miR-378a-3p** | 7.75 | 0.80 | 10.97 | 7.49 | 3.28 | 9.92 | 6.82 | 1.12 | 9.77 | 6.81 | 3.54 | 10.98 |
| **hsa-miR-20a-5p** | 1.21 | -0.09 | 3.65 | 1.15 | -0.90 | 2.62 | 1.15 | 0.21 | 2.17 | 0.90 | -0.54 | 2.22 |
| **hsa-miR-27b-3p** | -2.51 | -5.76 | -0.48 | -2.42 | -5.83 | -0.69 | -3.56 | -7.23 | -1.14 | -3.46 | -6.64 | -1.66 |
| **hsa-miR-140-5p** | -6.29 | -14.24 | -1.78 | -6.61 | -13.47 | -3.95 | -8.79 | -8.79 | -8.79 | . | . | . |
| **hsa-miR-25-3p** | -4.99 | -8.93 | -1.26 | -4.88 | -8.07 | -2.31 | -3.19 | -5.79 | -1.79 | -3.59 | -7.33 | -0.83 |
| **hsa-miR-23a-3p** | -3.14 | -5.68 | -1.31 | -3.04 | -4.85 | -1.45 | -4.42 | -7.61 | -2.64 | -4.70 | -11.77 | -3.45 |
| **hsa-let-7f-5p** | -4.06 | -7.30 | -1.31 | -3.51 | -12.76 | -0.62 | -5.18 | -10.75 | -2.14 | -4.57 | -11.30 | -2.41 |
| **hsa-miR-29a-3p** | -4.01 | -6.78 | -1.16 | -4.37 | -7.30 | -0.19 | -5.14 | -7.90 | -2.25 | -5.47 | -8.07 | -3.06 |
| **hsa-miR-195-5p** | -4.26 | -8.20 | -1.79 | -4.72 | -9.13 | -2.95 | -8.67 | -12.87 | -6.20 | -9.19 | -13.59 | -6.87 |
| **hsa-miR-205-5p** | 6.48 | -2.00 | 10.79 | 6.13 | -10.67 | 10.53 | 4.19 | -3.63 | 9.08 | 4.58 | -10.52 | 8.98 |
| **hsa-miR-214-3p** | -3.33 | -6.38 | 2.76 | -3.19 | -10.25 | 1.30 | -2.31 | -5.39 | 0.89 | -2.13 | -5.14 | 0.78 |
| **hsa-miR-372-3p** | -4.82 | -8.83 | -0.85 | -4.46 | -7.50 | 0.40 | -5.20 | -7.97 | -2.67 | -5.09 | -8.35 | -2.04 |
| **hsa-miR-103a-3p** | -4.91 | -14.43 | 0.87 | -4.82 | -12.39 | -0.44 | -4.68 | -10.46 | -0.80 | -5.22 | -12.64 | 1.24 |
| **hsa-miR-23b-3p** | -6.77 | -15.40 | -3.19 | -7.52 | -16.79 | -4.27 | -5.78 | -15.56 | -3.07 | -6.09 | -19.31 | -2.85 |
| **hsa-miR-122-5p** | -6.50 | -20.30 | -0.94 | -7.29 | -16.30 | -0.78 | -6.48 | -22.61 | -1.00 | -6.00 | -16.76 | -1.86 |
| **hsa-miR-373-3p** | -8.03 | -17.81 | -0.53 | -7.39 | -19.38 | 0.18 | -5.00 | -13.70 | 0.76 | -4.29 | -14.83 | 0.66 |
| **hsa-miR-200c-3p** | -8.61 | -15.60 | -5.80 | -6.98 | -10.57 | -2.05 | -9.65 | -16.52 | -5.58 | -6.37 | -14.61 | -0.15 |
| **hsa-miR-125b-5p** | -8.04 | -14.70 | -4.62 | -8.06 | -10.57 | -5.60 | -8.21 | -10.54 | -4.88 | -8.04 | -13.62 | -4.75 |
| **hsa-miR-140-3p** | . | . | . | . | . | . | -1.34 | -4.75 | 2.37 | -1.20 | -4.28 | 1.98 |
| **hsa-miR-152-3p** | . | . | . | . | . | . | -3.29 | -4.92 | -1.86 | -2.67 | -4.66 | -0.91 |
| **hsa-miR-199a/b-3p** | . | . | . | . | . | . | -1.43 | -4.28 | 0.14 | -2.25 | -5.18 | -0.10 |
| **hsa-miR-200b-3p** | . | . | . | . | . | . | -13.09 | -21.81 | -8.43 | -9.98 | -18.06 | -2.27 |
| **hsa-miR-182-5p** | . | . | . | . | . | . | -19.37 | -24.10 | -13.87 | -18.47 | -23.77 | -12.52 |

(.) missing values
