# Supplementary material for: Diagnostic plasma miRNA-profiles for ovarian cancer in patients with pelvic mass
Source: PLoS One. 2019 Nov 18;14(11):e0225249. doi: 10.1371/journal.pone.0225249 (PMC6860451; doi:10.1371/journal.pone.0225249)
Supplement: S3 Table — (DOCX) [file pone.0225249.s003.docx]

**S3 Table. Sensitivity and specificity rates of the markers on the discovery cohort**

|  | **sensitivity** | **specificity** |
| --- | --- | --- |
| **CA125** | 0.85 | 0.85 |
| **miR-221+miR200c** | 0.52 | 0.84 |
| **CA125+miR-221+miR200** | 0.91 | 0.84 |
