# Supplementary material for: Diagnostic plasma miRNA-profiles for ovarian cancer in patients with pelvic mass
Source: PLoS One. 2019 Nov 18;14(11):e0225249. doi: 10.1371/journal.pone.0225249 (PMC6860451; doi:10.1371/journal.pone.0225249)
Supplement: S1 Fig — (PDF) [file pone.0225249.s004.pdf]

Supplementary Figure S1

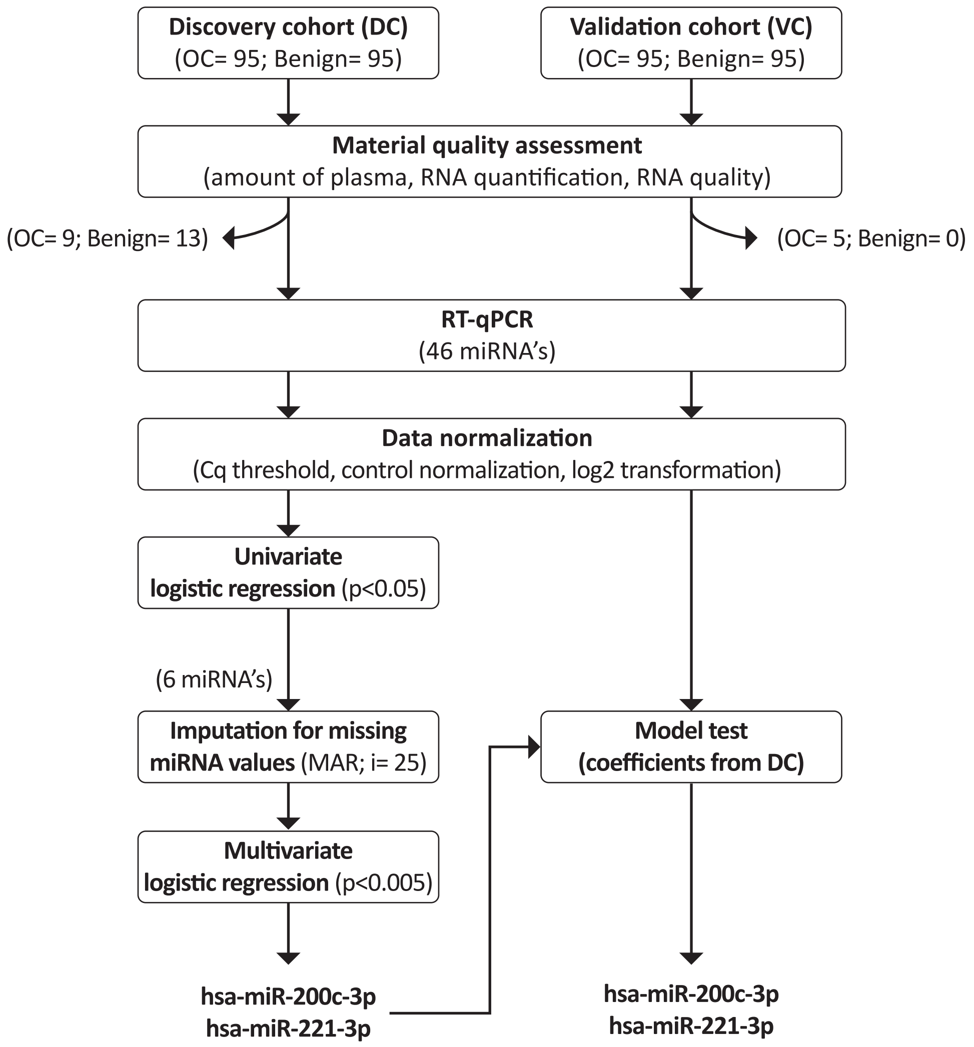

Supplementary Figure S1. Analysis pipeline for miRNA panel, in discovery and validation cohorts. Data handling and statistical analysis are described in the boxes, as well the output results following each step (outside the box). OC: ovarian cancer; DC: discovery cohort; VC: validation cohort; MAR: missing at random; i: number of imputations.
