# Supplementary material for: Diagnostic plasma miRNA-profiles for ovarian cancer in patients with pelvic mass
Source: PLoS One. 2019 Nov 18;14(11):e0225249. doi: 10.1371/journal.pone.0225249 (PMC6860451; doi:10.1371/journal.pone.0225249)
Supplement: S2 Fig — (PDF) [file pone.0225249.s005.pdf]

Supplementary Figure S2

A

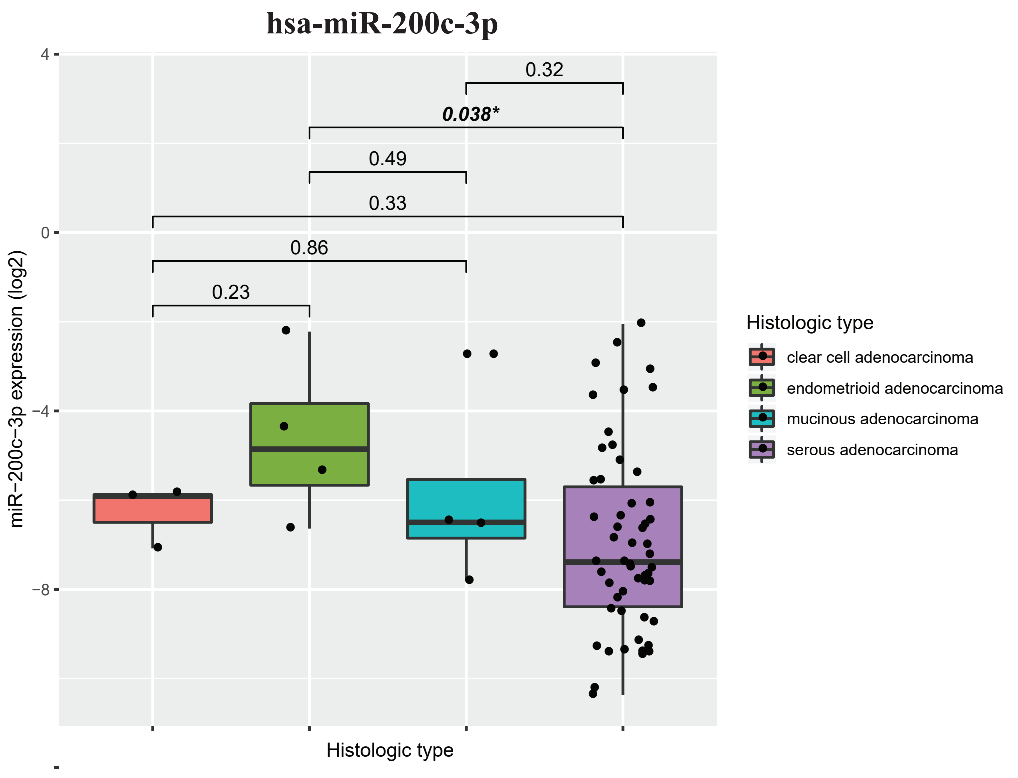

B

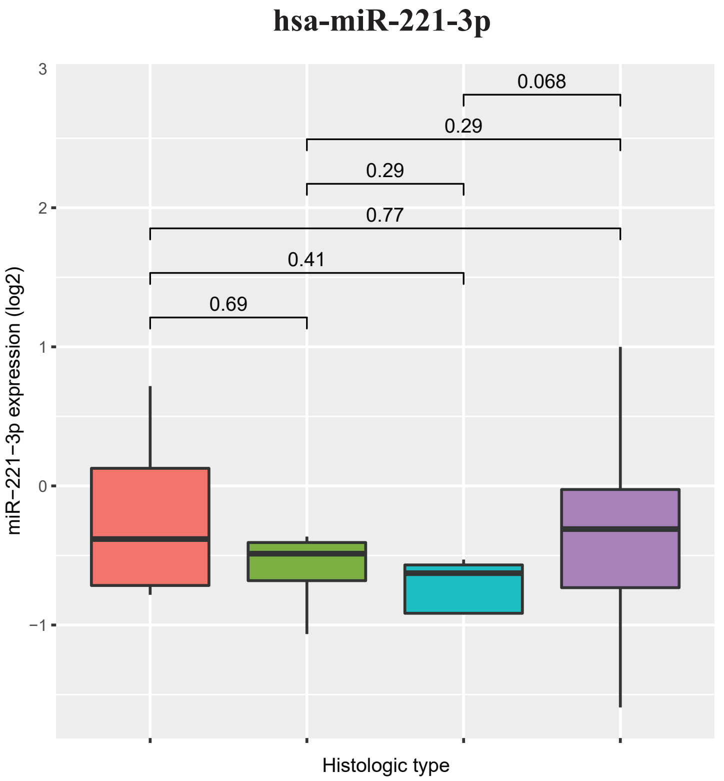

Supplementary Figure S2. miRNA expression in different histologic types. MiRNA expression (log2) for hsa-miR-200c-3p (A) and hsa-miR-221-3p (B) distributed for each histologic type from the discovery cohort. (*) p-value< 0.05
